# Low-Grade Appendiceal Mucinous Neoplasm Presenting as an Adnexal Mass

**DOI:** 10.1155/2017/7165321

**Published:** 2017-02-13

**Authors:** P. Panagopoulos, T. Tsokaki, E. Misiakos, V. Domi, C. Christodoulaki, D. Sioutis, N. Papantoniou

**Affiliations:** ^1^3rd Department of Obstetrics and Gynecology, National & Kapodistrian University of Athens, Medical School, Attikon University Hospital, Chaidari, Athens, Greece; ^2^3rd Department of Surgery, National & Kapodistrian University of Athens, Medical School, Attikon University Hospital, Chaidari, Athens, Greece

## Abstract

Appendiceal tumors are rare, late diagnosed neoplasms that may not be differentiated from adnexal masses even by advanced imaging methods and other diagnostic procedures. They may be asymptomatic and remain undiagnosed until surgery. We report a case of an 80-year-old postmenopausal woman presenting with a pelvic mass and a history of weight loss. The patient underwent laparotomy which revealed an appendiceal mucocele, for which she received a full oncological procedure. The histology report showed a low-grade appendiceal mucinous neoplasm, and the patient underwent six cycles of chemotherapy. Appendiceal tumors should be kept in mind in patients with adnexal mass.

## 1. Introduction

Neoplasms of the appendix are rare tumors accounting for about one percent of appendectomy specimens and 0.5 percent of intestinal neoplasms. Carcinoid tumors rank first (50%), followed by adenocarcinomas (8%) and mucinous cystadenomas of the appendix (7%) [1]. An appendiceal mucocele refers to a mucus-filled appendix and can be classified into the following histologic subtypes: mucosal hyperplasia, simple or retention cyst, mucinous cystadenoma, and mucinous cystadenocarcinoma [2].

These tumors are often discovered incidentally, either during a survey or at the time of surgery for other causes. Rupture of the mucocele, either spontaneously or accidentally during surgery, can lead to the development of pseudomyxoma peritonei, a condition in which malignant cells spread throughout the entire peritoneal cavity in the form of multiple mucinous implants on the peritoneal surfaces. The peritoneum is then seeded with mucus-producing cells, which continue to proliferate and produce mucus [3].

It is remarkable that appendiceal mucocele is not diagnosed in almost half of cases, and it is reported to be asymptomatic in 25% of cases. The most frequent symptom that raises the suspicion is sharp or persistent pain in the right lower abdominal quadrant [4, 5].

Herein we present a case of a postmenopausal woman with a pelvic mass and a history of weight loss. The patient underwent laparotomy which revealed an appendiceal mucocele, for which she received a full oncological procedure.

## 2. Case Report

**Figure 1:**
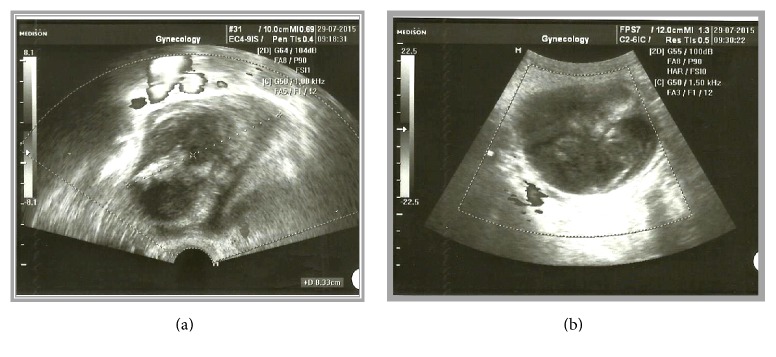
Transvaginal ultrasound showed a cystic formation sized 83 × 65 × 64 mm in the right adnexal area with signs of necrosis.

**Figure 2:**
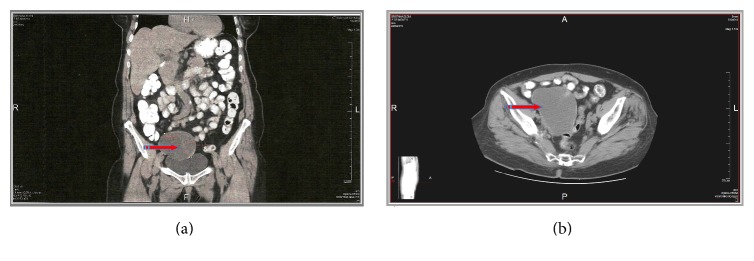
Abdominal CT revealed a cyst sized 100 × 80 mm with signs of calcification in the area of the right ovary (arrows).

An 80-year-old woman was referred to our clinic with a history of chronic abdominal pain and weight loss of 10 kilograms in the past six months. The patient was postmenopausal for 30 years and had a free medical history. Physical examination revealed no tenderness, but a hard and mobile mass palpable in the right iliac fossa. Further investigation by means of transvaginal ultrasound revealed a cystic formation, sized 83 × 65 × 64 mm in the right adnexal area. The mass was of mixed structure, comprising of solid and cystic areas. The wall was smooth and anechoic on ultrasound imaging. No papillary projections or septations were identified and there were no signs of blood flow during the color Doppler evaluation. The left ovary was illustrated measuring 25 × 13 × 11 mm, but the right ovary could not be visualized (Figures 1(a) and 1(b)). A polyp of the endometrium was also recorded, with total endometrial thickness of 8 mm. No free fluid was seen in the Douglas pouch. However, it was inconclusive whether the mass originated from the right adnexa or it concerned a uterine necrotic leiomyoma that protruded towards the adnexa. Because of the indeterminate ultrasound findings, an abdominal CT scan was carried out which identified a cyst sized 100 × 80 mm with signs of peripheral calcification attributed to a lesion of the right ovary (Figures 2(a) and 2(b)). As a routine investigation, when an ovarian tumor is suspected, our patient underwent colonoscopy, during which a benign polyp of the sigmoid colon was excised; also a submucosal round smooth cystic formation of one cm in diameter at the site of the appendix was identified. Tumor markers were within normal limits, with the exception of carcinoembryonic antigen (CEA: 54, 2 ng/mL).

The patient underwent laparotomy with the diagnosis of a pelvic mass, in which a cystic tumor originating from the appendix, sized 80 × 90 mm, was identified, whereas the uterus and ovaries were atrophic. The abdominal viscera were covered with surgical pad gauzes to protect from spillage of the cyst contents. However, several intestinal loops adhered to the cyst, and despite meticulous dissection, the cystic mass ruptured intraoperatively. Frozen section was performed, which revealed malignant mucinous neoplasm. Appendectomy, omentectomy, total abdominal hysterectomy, and bilateral salpingooophorectomy were performed, in collaboration with the General Surgery Team of the hospital. The histopathologic examination of the surgical specimen revealed a low-grade appendiceal mucinous neoplasm with focal invasion of the muscle layer (Stage I-T2N0M0, WHO Classification 2010), without desmoplastic reaction. The patient had an uneventful recovery and was discharged from the hospital one week later. Soon afterwards she underwent six cycles of chemotherapy and up to present, 12 months after surgery, she remains in good health.

## 3. Discussion

Appendiceal mucocele is considered very rare and usually presents as a distended, mucus-filled appendix. The course and prognosis of appendiceal mucocele is related to the histologic subtype. This tumor appears to have a slight female predominance and is usually diagnosed in patients in their fifth and sixth decades of life; however it may occur at any age [2, 6]. Neoplasms of the ovary, breast, kidney, or other tumors of the gastrointestinal tract can occur simultaneously in about 30% of patients [7–9].

Preoperative diagnosis of an appendiceal mucocele is difficult to accomplice because of the lack of specific symptoms. An appendiceal mucocele should be suspected in elderly women with the atypical finding of a mass originating from the adnexa in ultrasonography. This tumor may also represent an incidental finding during radiologic or endoscopic evaluation of nonspecific complaints. Elevated levels of tumor markers (e.g., carcinoembryonic antigen [CEA], CA 19-9) have been reported [10].

An abdominal CT scan can provide useful findings, such as a round or tubular cystic mass with calcification at the expected site of the appendix. We can also presume the existence of the tumor based on colonoscopic findings (smooth protruding mass arising from the appendiceal orifice) [11, 12]. However, a definitive diagnosis of an appendiceal mucocele is reached through pathological evaluation of the excised appendix.

Appendicitis, a mesenteric or duplication cyst, and an adnexal mass are to be considered in the differential diagnosis. In our case a diagnosis of a cyst originating from the right adnexa was initially established; however, an appendiceal mucocele was evident only at laparotomy. Perforation of the appendix or mucous extravasation at the time of surgery may happen in one-third of patients and this may lead to pseudomyxoma peritonei [7]. The proliferation of malignant cells throughout the peritoneal cavity can create mucinous ascites, which may lead to adhesion formation and possibly intestinal obstruction [13]. It appears that both the benign and malignant versions of appendiceal mucocele can generate pseudomyxoma peritonei; however this is more frequent and with worse prognosis in malignant cases [7, 9, 14, 15].

Open surgical resection constitutes the treatment of choice, even for a benign-appearing appendiceal mucocele, since lesions that appear to be benign on imaging studies may harbor a cystadenocarcinoma. Laparoscopic treatment should be avoided, as there is increased risk of rupture. In case of malignancy, routine oophorectomy should be performed at the time of surgery, since the ovaries represent a common organ for metastases [16, 17]. If there is spillage of mucin during surgery, a follow-up CT should be performed at one year postoperatively.

In conclusion, appendiceal mucocele is a rare finding in abdominal surgery. Ultrasonography and computerized tomography represent useful tools for diagnosis. However, diagnosis is often intraoperative and is based on histopathological examination. The course and prognosis of this rare tumor is related to the histologic subtype. Retention cysts, mucosal hyperplasia, or cystadenomas present with excellent survival rate (91 to 100%) after standard appendectomy. In patients with appendiceal cystadenocarcinomas, five-year survival has a wide range (6 to 100%), based on stage [15].
